# *Wuchereria bancrofti* transmission pattern in southern Mali prior to and following the institution of mass drug administration

**DOI:** 10.1186/1756-3305-6-247

**Published:** 2013-08-28

**Authors:** Yaya Ibrahim Coulibaly, Benoit Dembele, Abdallah Amadou Diallo, Sibylle Kristensen, Siaka Konate, Housseini Dolo, Ilo Dicko, Moussa Brema Sangare, Falaye Keita, Boakye A Boatin, Abdel Kader Traore, Thomas B Nutman, Amy D Klion, Yeya Tiemoko Touré, Sekou Fantamady Traore

**Affiliations:** 1 Mali International Center for Excellence in Research (ICER), University of Sciences, Techniques, and Technologies of Bamako (USTTB), Bamako, Mali; 2 Department of Epidemiology and International Health, University of Alabama at Birmingham, Birmingham, AL, USA; 3 Centre National d’Appui à la lutte contre la Maladie (CNAM), Bamako, Mali; 4 World Health Organisation, Geneva, Switzerland; 5 Laboratory of Parasitic Diseases, National Institute of Allergy and Infectious Diseases, National Institutes of Health, Bethesda, MD, USA

## Abstract

**Background:**

The Global Programme to Eliminate Lymphatic Filariasis (GPELF) was launched in 2000 with the goal of stopping transmission of lymphatic filariasis (LF) through yearly mass drug administration (MDA). Although preliminary surveys of the human population in Mali suggested that *Wuchereria bancrofti* infection was highly endemic in the Sikasso district, baseline entomological data were required to confirm high levels of transmission prior to the selection of villages in this region for a study of the impact of MDA on transmission of LF by anopheline vectors.

**Methods:**

*W. bancrofti* transmission was assessed in 2001 (pre-MDA) and 2002 (post-MDA) in the Central District of Sikasso in southern Mali by dissection of *Anopheles* mosquitoes caught using the human landing catch (HLC) method. The relative frequencies and molecular forms of *An. gambiae* complex were determined.

**Results:**

The majority (86%) of the anopheline vectors captured were identified as *An. gambiae* complex, and these accounted for >90% of the entomological inoculation rate (EIR) during both years of the study. There was a dramatic decrease in the number of *An. gambiae* complex mosquitoes captured and in the *An. gambiae* complex infectivity rates following MDA, accounting for the observed decrease in EIR in 2002 (from 12.55 to 3.79 infective bites per person during the transmission season). *An. funestus* complex mosquitoes were responsible for a low level of transmission, which was similar during both years of the study (1.2 infective bites per person during the transmission season in 2001 and 1.03 in 2002).

**Conclusions:**

Based on the entomological data from this study, the district of Sikasso was confirmed as an area of high *W. bancrofti* transmission. This led to the selection of this area for a multi-national study on the effects of MDA on LF transmission by anopheline vectors. Comparison of vector transmission parameters prior to and immediately following the first round of MDA demonstrated a significant decrease in overall transmission. Importantly, the dramatic variability in EIR over the transmission season suggests that the efficacy of MDA can be maximized by delivering drug at the beginning of the rainy season (just prior to the peak of transmission).

## Background

Lymphatic filariasis (LF) is a chronic debilitating infection caused by the mosquito-borne filarial nematodes, *Wuchereria bancrofti* (*W. bancrofti*), *Brugia malayi* and *Brugia timori.* Worldwide, more than one billion people are at risk of infection, among which 120 million are already infected, the majority of whom are in India, with an estimated 49.2% of the infection burden, followed by sub-Saharan Africa with 34.1% [1]. *W. bancrofti* is responsible for approximately 90% of LF cases worldwide and all of the cases in sub-Saharan Africa, where the most common vectors are *An. gambiae* and *An. funestus* complexes [2]. Forty-three million people are officially recognized as being disabled due to LF and millions more suffer from social and psychological problems [3]. Nevertheless, the socio-economic burden of LF is underestimated in many endemic areas that are among the poorest of the world [4].

In Mali, although the public health importance of LF was noted as early as the 1970′s [2], the prevalence and distribution were not studied again until 2002, when the National Lymphatic Filariasis Elimination Program (NLFEP) provided the first countrywide LF map. Based on antigen testing using ICT cards, all 8 administrative regions of Mali were shown to be endemic for LF with an overall prevalence of 7.07%, ranging from 1% in the north to 18.6% in the south. In anticipation of the launch of NLFEP activities in Mali, a pilot study of the impact of mass drug administration (MDA) with albendazole and ivermectin on *W. bancrofti* transmission was initiated in collaboration with the World Health Organization (WHO) in Ghana, Mali, and Nigeria. The present study was designed to provide baseline data on vector transmission in this highly endemic region of Mali and to assess the effects of the first round of MDA.

## Methods

### Study site identification and characterization

The study was initiated in the Sikasso district in southern Mali prior to the introduction of MDA for the elimination of LF. This area was historically known to be endemic for *W. bancrofti,* and mapping surveys performed by the National Program for the Elimination of Lymphatic Filariasis confirmed a high prevalence of circulating filarial antigen (CFA) positivity (as assessed by ICT card testing of 50-100 individuals/village) in the village of Dozanso and a neighboring village in 2001 (unpublished data). Additional ICT card surveys were subsequently carried out in the larger villages surrounding Dozanso in 2001 and led to the selection of 6 high prevalence villages (Dozanso, Gondaga Missasso, N’Torla, Niantansso and Zanadougou) for baseline entomological studies.

The study villages are comparable in terms of socio-cultural indicators, health care seeking behavior and disease perception. The distance between the villages and the community health care center of Kolokoba ranges from 6 to 15 km (mean 9.5 km), occupied by cotton fields, backwaters, and trees typical of the dense Sudan Savannah vegetation. Rainfall in this region ranges between 1200 and 1500 mm per year, with a rainy season that extends from July to December. Due to the high levels of transmission documented during the first year of the study, yearly MDA was instituted in the 6 study villages in June 2002, one month prior to the second entomological survey.

### Study population

A complete census, including the name, age, sex and profession of each inhabitant, was performed in all 6 villages. All dwellings were recorded and assigned an identification number. A global positioning system device (GPS) was used to produce basic maps of the locations of the 6 villages within the Central District of Sikasso. The total population of the 6 study villages was 3,681 in 2001, consisting primarily of farmers, whose main occupations are agriculture (cotton, maize, millet and peanut) and domestic animal breeding. The ICT card surveys carried out in 2001 revealed CFA prevalences varying from 81.8% in Niantanso (165/202) to 24.6% in Zanadougou (50/202) (Table 1). The prevalence of microfilaremia was assessed in 2002 (prior to the initiation of MDA) by examination of 3 slides of 20 μl of night blood/subject, and ranged from 40% in Dozanso (48/120) to 13.8% in N’Torla (27/196).

**Table 1:** Characteristics of the study population prior to MDA

**Villages**	**Total tested**	**Male**	**Female**	**Mf positive**	**CAg positive**
		**%**	**%**	**%**	**%**
Dozanso	120	54.2	45.8	40	61.7
Missasso	207	35.3	64.7	20.3	36.9
Gondaga	212	45.8	54.2	15.1	43.4
Niantanso	202	42.1	57.9	29.7	81.8
N’Torla	196	50.5	49.5	13.8	40.3
Zanadougou	202	30.7	69.3	17.3	24.6

*Mf* Microfilaremia, *CAg* Circulating filarial antigen.

A collective village-wide oral consent was obtained from the villages’ elders, and all study participants signed individual written informed consent. The study protocol and consent forms were approved by both the Institutional Review Board (IRB) of the World Health Organization/Tropical Diseases Research (WHO/TDR) and the ethics committee of the Faculty of Medicine, University of Bamako, Mali.

### Study design

This was a longitudinal study during which monthly entomological surveys were performed in 6 study villages from July to December in 2001 (prior to initation of MDA with albendazole and ivermectin) and in 2002 (one month after the first MDA).

### Laboratory analysis

Entomological surveys were performed 12 days per month (2 days/village/month) by the same team. Mosquitoes were collected by two trained field personnel in each room of four different houses in each village using the HLC method. One collection team worked from 6:00 pm to midnight and the second from midnight to 6:00 am. Mosquitoes were captured using a Colluzi and Petrarca type mouth aspirator connected to a paper cup as the storage container. A supervisor retrieved the containers at two-hour intervals. The captured mosquitoes were kept overnight at ambient temperature in a paper cup under a damp cloth and dissected the following morning.

Mosquitoes were sorted morphologically for species identification (*An. gambiae* and *An. funestus* complexes). Some *An. gambiae* complex specimens were processed by polymerase chain reaction (PCR) method to distinguish between the 2 members of the complex (*An. arabiensis* and *An. gambiae ss*). The *An. gambiae ss* were further processed by PCR to identify the molecular forms, M and S, as described by Favia *et al.*[5,6]. The head, thorax and abdomen were dissected separately for each mosquito and recovered parasite larvae were categorized into L1, L2 or L3 stages.

Entomologic parameters assessed included infection rate, infectivity rate, human biting rate (HBR) and entomological inoculation rate (EIR) and were calculated as previously described [5,7]:

- Infection rate: proportion of mosquitoes found infected after dissection with any *W. bancrofti* larval stage (L1–L3).

- Infectivity rate: proportion of mosquitoes found infected with one or more infective larvae (L3).

- Human biting rate (HBR): number of mosquitoes caught during the HLC × 30/(total number of collectors used per collection × number of collections in the month).

- Entomological inoculation rate (EIR): HBR × infectivity rate. The results of the monthly HBR (from all night HLC) multiplied by the *W. bancrofti* infectivity rate for a given species give an estimate of the number of infective bites of *W. bancrofti* received per human per month.

### Data management and analysis

Data were analyzed using SPSS version 14 (Statistical Package for Social Sciences) (SPSS Inc., Chicago, IL) and Prism V5.0 (GraphPad Software). The Chi square test or the Fisher’s exact test was used as appropriate to compare proportions. The confidence level was set at 95% for all statistical tests.

## Results

### Monthly variations in vector densities

A total of 23,265 and 12,986 mosquitoes were collected in the 6 villages of the district of Sikasso from July to December in 2001 and 2002, respectively. Overall, *An. gambiae* complex (20,957 in 2001 and 11,190 in 2002) were more frequently captured than *An. funestus* complex (2,308 in 2001 and 1,796 in 2002) among the active vector fauna. At the beginning of the transmission season, *An. gambiae* complex was collected more frequently than *An. funestus* complex (158 fold more in July 2001 and 138 fold more in August 2002). This trend diminished towards the end of the transmission season with equal collection of both species in December 2001 (Table 2) and only a two-fold increase in collection of *An. gambiae* complex in November and December 2002 (Table 3).

**Table 2:** Monthly variation of the entomological parameters for the transmission of lymphatic filariasis in six villages of the District of Sikasso in 2001

***Anopheles funestus *complex**						
**Month**	**Collected**	**Dissected**	**Infected (%)**	**Infective (%)**	**HBR**	**EIR**
July	25	25	0 (0)	0 (0)	4	0
Aug	33	33	0 (0)	0 (0)	5	0
Sep	278	148	4 (2.7)	2 (1.4)	43	0.6
Oct	1402	789	51 (6.5)	15 (1.9)	219	4.2
Nov	514	432	17 (3.9)	13 (3)	80	2.4
Dec	56	44	0 (0)	0 (0)	9	0
**Total**	**2,308**	**1471**	**72 (4.9)**	**30 (2)**	**60**	**1.2**
***Anopheles gambiae *complex**						
**Month**	**Collected**	**Dissected**	**Infected (%)**	**Infective (%)**	**HBR**	**EIR**
July	3960	3959	123 (3.1)	88 (2.2)	618.75	13.75
Aug	4971	4948	137 (2.8)	91 (1.8)	776.72	14.28
Sep	9096	4708	211 (4.5)	120 (2.5)	1421.25	35.53
Oct	2320	2005	137 (6.8)	61 (3)	362.5	10.9
Nov	544	544	36 (6.6)	12 (2.2)	85	1.88
Dec	66	66	2 (3)	0 (0)	10.31	0
**Total**	20,957	16,230	646 (4)	**372 (2.3)**	545.76	12.55

*N* Number, *%* Percent, *HBR* Monthly biting rate, *EIR* Entomological inoculation rate.

**Table 3:** Monthly variation in the entomological parameters related to the transmission of lymphatic filariasis in six villages of the District of Sikasso in 2002

***Anopheles funestus *complex**						
**Month**	**Collected**	**Dissected**	**Infected (%)**	**Infective (%)**	**HBR**	**EIR**
July	14	14	2 (14.3)	0 (0)	2.2	0
Aug	18	18	2 (11.1)	0 (0)	2.8	0
Sep	342	342	22 (6.4)	4 (1.2)	53.4	0.64
Oct	786	786	38 (4.8)	16 (2)	122.8	2.46
Nov	600	600	26 (4.3)	20 (3.3)	93.8	3.1
Dec	36	36	2 (5.6)	0 (0)	5.6	0
**Total**	**1,796**	**1,796**	**92 (5.1)**	**40 (2.2)**	**46.8**	**1.03**
***Anopheles gambiae *complex**						
**Month**	**Collected**	**Dissected**	**Infected (%)**	**Infective (%)**	**HBR**	**EIR**
July	1,646	1,646	18 (1.1)	2 (0.1)	257.2	0.26
Aug	2,488	2,488	37 (1.5)	5 (0.2)	388.8	0.78
Sep	2,846	2,846	244 (8.6)	40 (1.4)	444.7	6.23
Oct	3,214	3,214	160 (5)	70 (2.2)	502.2	11.05
Nov	924	924	34 (3.7)	22 (2.4)	144.4	3.46
Dec	72	72	12 (16.7)	2 (2.8)	11.3	0.31
**Total**	**11,190**	**11,190**	**505 (4.5)**	**141 (1.3)**	**291.4**	**3.79**

*N* Number, *%* Percent, *HBR* Monthly biting rate, *EIR* Entomological inoculation rate.

Relative frequencies of An. gambiae complex members and An. gambiae s.s. molecular forms.

Among the 15,869 *An. gambiae* complex members examined by PCR for specific species identification, 99.02% (15,713/15,869) were *An. gambiae s.s.* and 0.98% (156/15,869) was *An. arabiensis*. The frequency of *An. gambiae s.s.* decreased towards the end of the rainy season (December) while that of *An. arabiensis* increased slightly (Trends Chi square = 90.57; p < 10^-4^) (Table 4). Significant monthly variation in the relative frequencies of the two species was observed (p < 10^-6^). The overwhelming majority (95.09%; 14,942/15,713) of the *An. gambiae s.s.* collected in 2001 were the S molecular form (Table 4). This high frequency of the S molecular form was observed in all of the study villages (data not shown).

**Table 4:** **Monthly variation in the relative frequencies of *Anopheles gambiae complex members *and the molecular forms of *Anopheles gambiae sensu stricto *in 2001**

	***An. gambiae *complex members**				***An. gambiae ss *molecular forms**			
	*** An. gambiae ss***		*** An. arabiensis***		** M Form**		** S Form**	
Months	N	(%)	N	(%)	N	(%)	N	(%)
July	3,895	99.7	12	0.3	195	5	3,700	95
August	4,859	99.5	24	0.5	233	4.8	4,626	95.2
September	4,480	98.6	63	1.4	211	4.7	4,269	95.3
October	1,895	98.4	31	1.6	80	4.2	1,815	95.8
November	521	95.8	23	4.2	31	6	490	94
December	63	95.5	3	4.5	21	33.3	42	66.7
Total	15,713	99.02	156	0.98	771	4.91	14,942	95.09

S Form = *An. gambiae* Form Bamako or Savannah; M Form = *An. gambiae* Form Mopti.

### Vector infection rates and transmission pattern

**Figure 1:**
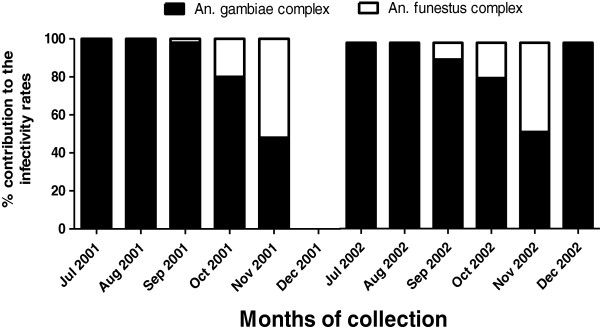
**Species contribution to the overall monthly infectivity rates in 2001 and 2002.** The black represents the contribution of *Anopheles gambiae* complex and the white the contribution of *Anopheles funestus* complex.

Both *An. gambiae* and *An. funestus* complexes were found to be harboring infective larvae during the two years of study (2001 and 2002). In July and August, 100% of the infective *W. bancrofti* larvae were recovered from *An. gambiae* complex (Figure 1). *An. funestus* complex harbored infective larvae with increasing rates from September to November. No infective mosquito was recovered in December 2001 (Table 2). In both 2001 and 2002, the *An. funestus* complex became increasingly more important in LF transmission from September through November (Table 2 and 3).

Overall, infection rates were comparable between *An. gambiae* and *An. funestus* complexes with 4% versus 4.9% (Chi^2^ = 2.90; p = 0.09) in 2001 (Table 2) and 4.5% versus 5.1% (Chi^2^ = 1.31; p = 0.25) in 2002 (Table 3). In 2001, *An. funestus* complex was found to be carrying *W. bancrofti* larvae from September to November with monthly infection rates ranging from 2.7% to 6.5% while infected *An. gambiae* complex was recovered each month from July to December with rates ranging from 2.8% to 6.8% (Table 2). In 2002, both *An. funestus* and *An. gambiae* complexes were found infected from July to December with monthly rates ranging respectively from 4.3% to 14.3% and 1.1% to 16.7% (Table 3). Whereas infection and infectivity rates were similar in 2001 and 2002 for *An. funestus* complex (4.9% versus 5.1% infection; Chi^2^ = 0.04, p = 0.82 and 2% versus 2.2% infectivity; Chi^2^ = 0.06, p = 0.80), the infectivity rate for *An. gambiae* complex significantly decreased in 2002 following MDA (1.3% versus 2.3% infectivity in 2001; Chi^2^ = 37.86, p < 10^-3^), despite a small increase in infection rate (4.5% versus 4.0% infection in 2001; Chi^2^ = 4.54, p = 0.03).

In 2001, the overall monthly EIR was more than 10 fold higher for *An. gambiae* complex than *An. funestus* complex*.* The *An. gambiae* complex was responsible for 0 to 35.53 infective bites per human per month. The *An. funestus* complex was responsible of 0 to 4.2 infective bites per human per month. In November 2001, the *An. funestus* complex was responsible for more infective bites than the *An. gambiae* complex (2.4 versus 1.8) but no infective bite was recorded in December (Table 2). In 2002, there was a dramatic decrease in the overall EIR for *An. gambiae* complex as compared to 2001 (from 12.55 to 3.79 infective bites per person during the transmission season). In contrast, for *An. funestus* complex, the overall EIR in 2001 (1.2 infective bite/person) (Table 2) was similar to that recorded in 2002 (1.03 infective bite/person) (Table 3).

## Discussion

The baseline entomological data collected in this longitudinal study confirmed measurable transmission of *W. bancrofti* in the 6 study villages in Sikasso prior to the initiation of MDA. As had been reported previously in Mali, *An. gambiae* and *An. funestus* complexes were the predominant vectors [2,8]. In keeping with the high prevalence of human infection in these villages, the recorded vector densities were higher, especially for *An. gambiae* complex, than those reported in Banambani (Sudan savannah area) in Mali, where *W. bancrofti* is endemic but the prevalence of infection is lower [8]. Other *Anopheles* species (*An. pharaoensis*, *An. nili*, *An. rufipes*) were collected but not systematically processed during this study because of their very low relative frequencies, precluding an epidemiologically significant role in the transmission of LF, and the fact that they have not been recognized as vectors of *W. bancrofti* in Mali and other neighboring West African countries [2,9].

Among the *An. gambiae* complex members examined by PCR for specific species identification, the S form of *Anopheles gambiae s.s*. was predominant. A predominance of the S form of *An. gambiae s.s* among vectors of LF has also been observed in Ghana [10]. Although the PCR identification of *An. gambiae* complex species and *An. gambiae s.s.* molecular forms was not performed on all the collected mosquitoes for logistical reasons, at least 76% of the mosquitoes collected each month were dissected to ensure that the samples tested were temporally and geographically representative. Mosquitoes were sent for PCR analyses without identity numbers that could link them to the dissection results precluding the determination of infection rates for the different molecular forms.

Overall, the highest monthly vector relative frequencies for *An. gambiae* complex were found in July and August (at least 99% of the vectors collected in the month), while those of *An. funestus* complex were observed in November and December (at least 33% of the vectors collected in the month). Similar variations in the relative frequencies of the two vectors were reported in Banambani [8] and are related to differences in environmental conditions during the transmission season and the breeding preferences of each species (*An. funestus* complex prefers shadowed and vegetated breeding sites while *An. gambiae* complex prefers sunny breeding sites with limited vegetation) [2,8]. The frequencies of *An. gambiae s.s.* and *An. arabiensis*, two members of the *An. gambiae* complex, also showed differing patterns during the transmission season.

Due to the low infection and infectivity rates, processing of pools of *Anopheles* vectors for *W. bancrofti* infection is the recommended strategy for following vector transmission rates during MDA [11,12]. A recently developed L3 specific RT-PCR allows infective pools to be distinguished from infected pools and provides a more accurate determination of the transmission potential for *W. bancrofti*[9,13]. In the present study, the infection and infectivity profiles of the two morphologically distinct *Anopheles* species complexes (*An. gambiae s.l.* and *An. funestus*) were quite different, suggesting that the two species complexes should be processed for PCR in separate pools if detailed information regarding their relative contributions to monthly transmission is desired. Nonetheless, in the setting of post MDA assessment, where human-vector contact is the main factor of interest, *An. gambiae* and *An. funestus* complexes can be processed in the same pool [11,13].

In 2002 (post MDA), the number of mosquitoes caught was approximately half that in 2001 (before MDA). This effect was most dramatic for *An. gambiae* complex where the number captured decreased by almost 50%. Potential reasons for this decrease in mosquito numbers include changes in climate, increased awareness of the study area population with respect to the role of mosquitoes in disease transmission (resulting in less breeding sites and increased use of insecticide treated nets), and the effect of ivermectin on mosquito survivorship. Examination of the rainfall, temperature and humidity records for the region did not show any major differences between 2001 and 2002, suggesting that climate did not play a major role in the decreased number of mosquitoes. Although decreases in mosquito numbers following the initiation of MDA [14] and an effect of ivermectin on mosquito survivorship [15,16] have both been described, these factors were not directly addressed in the present study.

Whereas the decreased vector numbers in 2002 (post-MDA) clearly contributed to the overall decrease in EIR observed for the *An. gambiae* complex, infectivity also declined significantly in 2002, suggesting that multiple factors may have played a role in the observed decrease in transmission including the decrease of the mf prevalence and loads consecutive to the MDA [14]. The fact that a similar decrease in EIR was not seen for *An. funestus* complex may have been due to the low overall numbers of *An. funestus* complex mosquitoes captured, although a higher degree of facilitation by *An. funestus* complex as compared to *An. gambiae* complex cannot be excluded. Unfortunately, the study was not designed to address this issue, and published data comparing facilitation between the two species are limited [17,18].

Despite the fact that the overall mosquito infection rates were relatively stable during the six months of collection in each of the two transmission seasons, the EIR for *W. bancrofti* varied considerably over the course of the seasons as a result of the large differences in vector densities and HBR [11]. This has important implications for the timing of MDA for LF in this region, since drug administration conducted at the beginning of the rainy season would be predicted to be most effective in decreasing transmission due to maximal reduction in mf prevalence and loads at the precise time that vector density and biting rates are beginning to rise.

Ethical approval for this study was obtained from WHO and University of Bamako. At the time that the study was performed, Human Landing Catch was considered an ethically acceptable method of mosquito collection. The collectors in this study were adult village residents normally exposed to mosquito bites. The collectors were not given antimalarial prophylaxis, but were provided access to a health practitioner (nurse) during the study in the event of malaria infection as recommended for adult subjects living in malaria endemic area. Since the goal of HLC is to collect the mosquito before it bites, the risk of infective bite is actually quite low. Although HLC is still used in some settings, research is actively ongoing in our center and others to find a comparable method that does not involve human bait [19-21].

## Conclusions

In conclusion, the entomological data from the present study confirmed the district of Sikasso as an area of high *W. bancrofti* transmission. This led to the selection of this area as the site of a multi-national study on the effects of MDA on LF transmission by anopheline vectors and as the first region in Mali for implementation of MDA with ivermectin and albendazole to eliminate transmission of LF. Comparison of the vector transmission parameters prior to and immediately following the first round of MDA demonstrated a significant decrease in overall transmission after institution of MDA. Importantly, the dramatic variability in EIR over the transmission season suggests that the efficacy of MDA can be maximized by delivering drug at the beginning of the rainy season (just prior to the peak of transmission).

## Competing interests

The authors declare that they have no competing interests.

## Authors’ contributions

YIC, SFT, YTT designed and conceived the study; TBN, ADK, BAB, YTT approved final version of the manuscript and helped with the analysis and drafting of the manuscript; YIC, BD, AAD, SKo, FK, AKT collected and processed the samples and drafted the manuscript; YIC, BD, HD, ID, MBS, SK managed the data, did the statistical analysis and helped to draft the manuscript. All the authors read and approved the final manuscript.
